# Common Biomarkers of Endothelial Dysfunction Across Highly Prevalent Diseases with Cardiovascular Risk: Functional Characterization and Prognostic Implications

**DOI:** 10.3390/ijms27093829

**Published:** 2026-04-25

**Authors:** Julia Martinez-Sanchez, Sergi Torramadé-Moix, Ana Belén Moreno-Castaño, Erica Lafoz, Jordi Rovira, Fritz Diekmann, Lida Maria Rodas, Elena Cuadrado-Payán, Isabel Galceran, Aleix Cases, Ana Paula Dantas, Joan Albert Barberà, Olga Tura-Ceide, Fàtima Crispi, Eduard Gratacós, Héctor García-Calderó, Juan Carlos García-Pagán, Virginia Hernández-Gea, Gines Escolar, Arturo Pereira, Maribel Diaz-Ricart

**Affiliations:** 1 Hemostasis and Erythropathology Laboratory, Hematopathology, Department of Pathology, Centre de Diagnòstic Biomèdic (CDB), Hospital Clínic de Barcelona, Institut d’Investigacions Biomèdiques August Pi i Sunyer (IDIBAPS), Universitat de Barcelona, 08036 Barcelona, Spain; 2 Barcelona Endothelium Team, 08036 Barcelona, Spain; 3 Barcelona Hepatic Hemodynamic Laboratory, Liver Unit, Hospital Clínic de Barcelona, Institut d’Investigacions Biomèdiques August Pi i Sunyer (IDIBAPS), 08036 Barcelona, Spain; 4 Laboratori Experimental de Nefrologia i Trasplantament (LENIT), IDIBAPS, 08036 Barcelona, Spain; jrovira1@recerca.clinic.cat (J.R.);; 5 RICORS 2040 (RD21-0005-0003), Instituto de Salud Carlos III, 28029 Madrid, Spain; 6 Department of Nephrology and Kidney Transplantation, Institut Clinic de Nefrologia i Urologia (ICNU), Hospital Clínic de Barcelona, Fundació de Recerca Clínic Barcelona-IDIBAPS, Universitat de Barcelona, 08036 Barcelona, Spainigalceran@clinic.cat (I.G.); acases@clinic.cat (A.C.); 7 Department of Cardiology, Hospital Clínic de Barcelona, Institut d’Investigacions Biomèdiques August Pi i Sunyer (IDIBAPS), Universitat de Barcelona, 08036 Barcelona, Spain; 8 Department of Pulmonary Medicine, Hospital Clínic de Barcelona, Institut d’Investigacions Biomèdiques August Pi i Sunyer (IDIBAPS), Universitat de Barcelona, 08036 Barcelona, Spain; 9 Biomedical Research Networking Centre on Respiratory Diseases (CIBERES), 28029 Madrid, Spain; 10 Translational Research Group on Cardiovascular Respiratory Diseases (CAREs), Dr. Josep Trueta University Hospital de Girona, Santa Caterina Hospital de Salt and the Girona Biomedical Research Institute (IDIBGI-CERCA), 17007 Girona, Spain; 11 BCNatal|Fetal Medicine Research Center (Hospital Clínic and Hospital Sant Joan de Déu), Institut d’Investigacions Biomèdiques August Pi i Sunyer (IDIBAPS), Universitat de Barcelona, 08036 Barcelona, Spain; 12 Centre for Biomedical Research on Rare Diseases (CIBER-ER), 28029 Madrid, Spain; 13 Centro de Investigación Biomédica en Red de Enfermedades Hepáticas y Digestivas (CIBERehd), Instituto de Salud Carlos III, 28029 Madrid, Spain; 14 Health Care Provider of the European Reference Network on Rare Liver Disorders (ERN-Liver), University of Barcelona, 08036 Barcelona, Spain; 15 Centre de Diagnòstic Biomèdic (CDB), 08036 Barcelona, Spain

**Keywords:** endothelial cells, cardiometabolic diseases, endothelial dysfunction biomarkers, apixaban, EUK134

## Abstract

Endothelial dysfunction (ED) arises in multiple pathologies, and its severity correlates with disease progression. Common ED biomarkers could provide prognostic value for associated complications. This study aims to identify shared ED biomarkers and assess their prognostic significance. Endothelial cells in culture (human microvascular endothelial cells, HMEC-1) were exposed to sera from patients in five disease groups (*n* = 20 patients/group)—liver cirrhosis with portal hypertension, idiopathic pulmonary arterial hypertension, placental disorders such as intrauterine growth restriction, coronary artery disease with acute myocardial infarction, and chronic kidney disease—or matched controls, in the absence/presence of anti-inflammatory (apixaban) and antioxidant (EUK134) compounds. We explored changes in: VCAM-1, ICAM-1, eNOS, VWF, extracellular matrix thrombogenicity, and reactive oxygen species (ROS). In serum samples, proteomics and metabolomics analyses (including lipids, amino acids, and polar metabolites) were performed through an extraction protocol to identify common ED biomarkers. Expression of VCAM-1, ICAM-1, VWF, platelet adhesion, and ROS increased in most groups versus controls (*p* < 0.05). Both drugs decreased all biomarker levels except eNOS (*n* = 6 for in vitro experiments). For serum ED biomarkers, 18 metabolites and 24 proteins showed AUC-ROC and hit rates >77.5%, and six metabolites were associated with event-free survival. These diseases share ED driven by systemic inflammatory, oxidative, and metabolic stress, are partially reversible in vitro, and are linked to biomarkers associated with clinical outcomes. Overall, ED emerges as a modifiable pathological axis with potential prognostic value.

## 1. Introduction

The endothelium is a dynamic biological interface between circulating blood and underlying tissues, playing a central role in regulating vascular homeostasis, including the balance between vasoconstriction and vasodilation, coagulation and fibrinolysis, and cellular proliferation and apoptosis [1]. Endothelial cells (ECs) possess a remarkable capacity to adapt to a wide range of local physiological and pathological stimuli. However, sustained exposure to chronic stimuli or acute pathological insults may induce maladaptive endothelial responses, ultimately leading to functional impairment and structural alterations.

Endothelial dysfunction (ED) is defined as a dysregulation of normal endothelial functions and is characterized by reduced vasodilatory capacity, increased proinflammatory and prothrombotic activity, and abnormal regulation of vascular growth and remodeling. ED has been documented in a broad spectrum of clinical conditions, including liver cirrhosis with portal hypertension (LCPH) [2,3,4], idiopathic pulmonary arterial hypertension (IPH) [5,6], placental disorders such as intrauterine growth restriction (IGR) [7,8,9], coronary artery disease with acute myocardial infarction (AMI) [10], stages 4–5 of chronic kidney disease (CKD) [11], and other related pathologies. Across these disease states, ED is generally attributed to an imbalance between vasodilator and vasoconstrictor pathways, accompanied by a pathological vascular remodeling [12].

Despite the well-established role of ED in the pathogenesis of several diseases [13], the mechanisms underlaying its initiation and persistence remain incompletely understood. Experimental studies in animal models of ED have identified several molecular and pathophysiological alterations that, when specifically targeted, result in significant improvement of endothelial function [14,15,16,17,18]. Nevertheless, the successful translation of these preclinical findings into clinical practice has been limited. Moreover, to date, no reliable biomarkers have been fully validated for the identification, stratification, or assessment of ED severity in clinical settings.

This translational gap between experimental models and patients with ED may be partly explained by the marked phenotypic heterogeneity of ECs, which is determined not only by their anatomical location but also by local environmental cues and epigenetic regulatory mechanisms. Consequently, identifying shared pathophysiological pathways (inflammation, increased thrombogenicity, and oxidative stress) and common circulating biomarkers across different forms of ED could facilitate earlier diagnosis and support the development of novel therapeutic strategies to prevent or reverse ED and its deleterious effects across multiple disease contexts.

Therapeutic strategies targeting the endothelium and modulating endothelial responses may represent a promising approach for the prevention and treatment of ED. In this context, apixaban (APIX), a factor Xa inhibitor with reported anti-inflammatory properties beyond its anticoagulant effects [19,20], and EUK134, a synthetic compound with catalase and superoxide dismutase (SOD) mimetic activity and antioxidant effect [21], have been proposed as potential modulators of ED.

It should also be noted that any in vivo research into potential treatments for ED will require readily available serum biomarkers that enable both the identification of prospective candidates and the assessment of treatment response.

To gain further insight into the ED associated with the aforementioned diseases, we studied a cohort of patients diagnosed with LCPH, IPH, AMI, and CKD and pregnant women with IGR. The objectives of this study were to: (1) characterize in vitro ED induced by patients’ sera compared with healthy controls; (2) evaluate the inhibitory effects of a factor Xa inhibitor and a SOD mimetic; and (3) identify potential circulating biomarkers of ED through large-scale metabolomic and proteomic analyses.

## 2. Results

### 2.1. Expression of Inflammatory Biomarkers on the Surface of ECs

Levels of the adhesion molecules vascular cell adhesion protein 1 (VCAM-1) and intercellular adhesion molecule (ICAM-1) were significantly increased (*p* < 0.01) in cultures supplemented with sera from patients with LCPH, IPH, CKD, and IGR but not in those supplemented with sera from patients with AMI, compared with controls, either healthy individuals (CONTROL) or healthy pregnant women (IGR CONTROL) (Supplementary Table S1).

**Figure 1:**
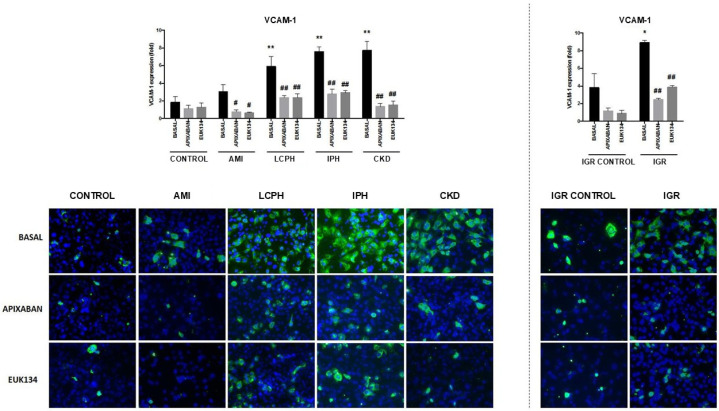
Representative immunofluorescence images (400×) of VCAM-1 (green) expression on endothelial cells (ECs). Nuclei were stained with DAPI (blue). ECs were exposed to serum from healthy donors (CONTROL), and from patients suffering from coronary artery disease with acute myocardial infarction (AMI), liver cirrhosis with portal hypertension (LCPH), idiopathic pulmonary arterial hypertension (IPH), and chronic kidney disease (CKD) and pregnant women with intrauterine growth restriction (IGR). Results from the latest group were compared with those obtained with sera from a control group of pregnant women (IGR CONTROL). Studies were performed in the absence (BASAL) and presence of apixaban and EUK134. Bar diagrams show VCAM-1 expression, with fold increases relative to results obtained by exposing cells to a commercial human serum (Mean ± SEM, *n* = 6). * *p* < 0.05 and ** *p* < 0.01 compared to the control group (CONTROL). # *p* < 0.05 and ## *p* < 0.01 compared to the respective basal state of each group (BASAL).

**Figure 2:**
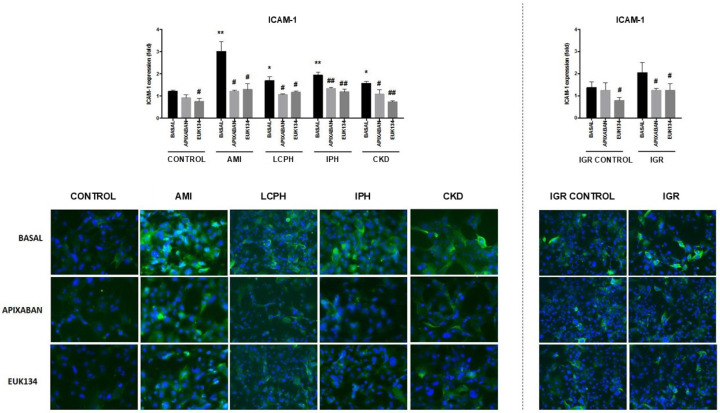
Representative immunofluorescence images (400×) of ICAM-1 (green) expression on endothelial cells (ECs). Nuclei were stained with DAPI (blue). ECs were exposed to serum from healthy donors (CONTROL), and from patients suffering from coronary artery disease with acute myocardial infarction (AMI), liver cirrhosis with portal hypertension (LCPH), idiopathic pulmonary arterial hypertension (IPH), and chronic kidney disease (CKD) and pregnant women with intrauterine growth restriction (IGR). Results from the latest group were compared with those obtained with sera from a control group of pregnant women (IGR CONTROL). Studies were performed in the absence (BASAL) and presence of apixaban and EUK134. Bar diagrams show ICAM-1 expression, with fold increases relative to results obtained by exposing cells to a commercial human serum (Mean ± SEM, *n* = 6). * *p* < 0.05 and ** *p* < 0.01 compared to the control group (CONTROL). # *p* < 0.05 and ## *p* < 0.01 compared to the respective basal state of each group (BASAL).

When experiments were performed in the presence of apixaban or EUK134, a significant reduction in both VCAM-1 and ICAM-1 expression was observed in all groups compared to the absence of drugs (Supplementary Table S2, Figure 1 and Figure 2).

### 2.2. Thrombogenicity of Extracellular Matrices Generated by ECs

Regarding thrombogenicity outcomes, both the expression of the Von Willebrand Factor (VWF) and the platelet-covered surface under flow were significantly increased in EC co-cultured with sera from patients with the five conditions under study, compared with both control groups (Supplementary Table S1).

**Figure 3:**
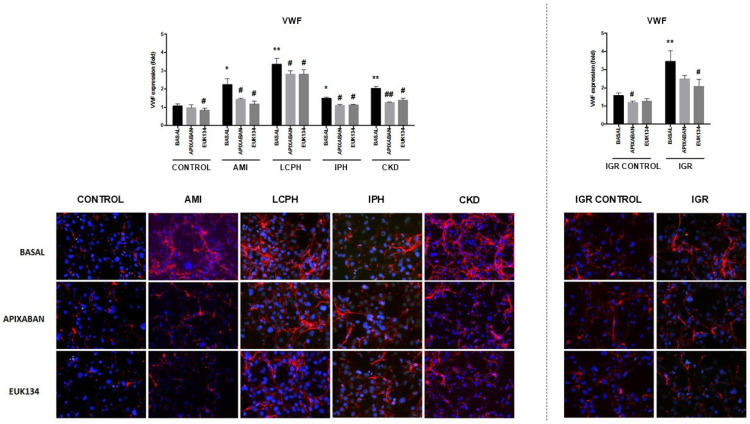
Representative immunofluorescence images (400×) of VWF (red) expression in endothelial cells (ECs). Nuclei were stained with DAPI (blue). ECs were exposed to serum from healthy donors (CONTROL), and from patients suffering from coronary artery disease with acute myocardial infarction (AMI), liver cirrhosis with portal hypertension (LCPH), idiopathic pulmonary arterial hypertension (IPH), and chronic kidney disease (CKD) and pregnant women with intrauterine growth restriction (IGR). Results from the latest group were compared with those obtained with sera from a control group of pregnant women (IGR CONTROL). Studies were performed in the absence (BASAL) and presence of apixaban and EUK134. Bar diagrams show VWF expression, with fold increases relative to results obtained by exposing cells to a commercial human serum (Mean ± SEM, *n* = 6). * *p* < 0.05 and ** *p* < 0.01 compared to the control group (CONTROL). # *p* < 0.05 and ## *p* < 0.01 compared to the respective basal state of each group (BASAL).

**Figure 4:**
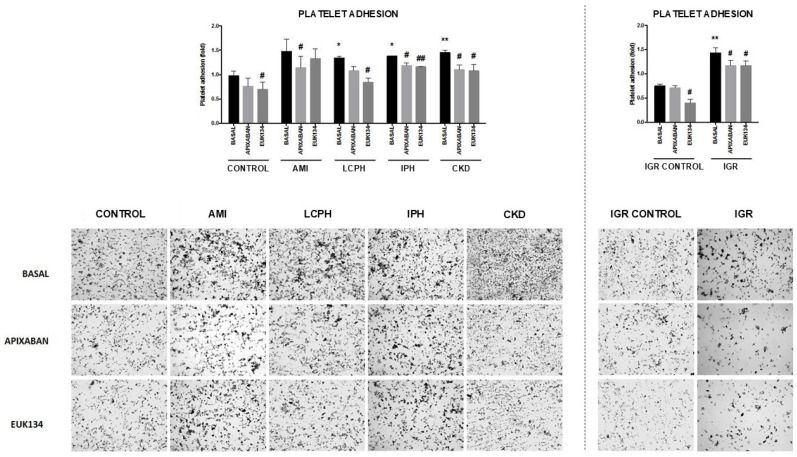
Representative images (400×) showing platelet adhesion (gray spots) on the extracellular matrices (ECM) generated by endothelial cells (ECs) exposed to different serum samples: from healthy donors (CONTROL), and from patients suffering from coronary artery disease with acute myocardial infarction (AMI), liver cirrhosis with portal hypertension (LCPH), idiopathic pulmonary arterial hypertension (IPH), and chronic kidney disease (CKD) and pregnant women with intrauterine growth restriction (IGR). Results from the latest group were compared with those obtained with sera from a control group of pregnant women (IGR CONTROL). ECM were exposed to circulating citrated blood, by means of a parallel-plate perfusion chamber (800 s^−1^, 5 min). Studies were performed in the absence (BASAL) and presence of apixaban and EUK134. Bar diagrams show the surface covered by platelets (platelet adhesion) as fold increases relative to results obtained by exposing cells to a commercial human serum (Mean ± SEM, *n* = 6). * *p* < 0.05 and ** *p* < 0.01 compared to the control group (CONTROL). # *p* < 0.05 and ## *p* < 0.01 compared to the respective basal state of each group (BASAL).

In the presence of apixaban and EUK134, VWF expression and the surface covered by platelets decreased significantly in all study conditions compared with the absence of drugs (Supplementary Table S2, Figure 3 and Figure 4).

### 2.3. Oxidative State in ECs Exposed to the Patients’ Sera

**Figure 5:**
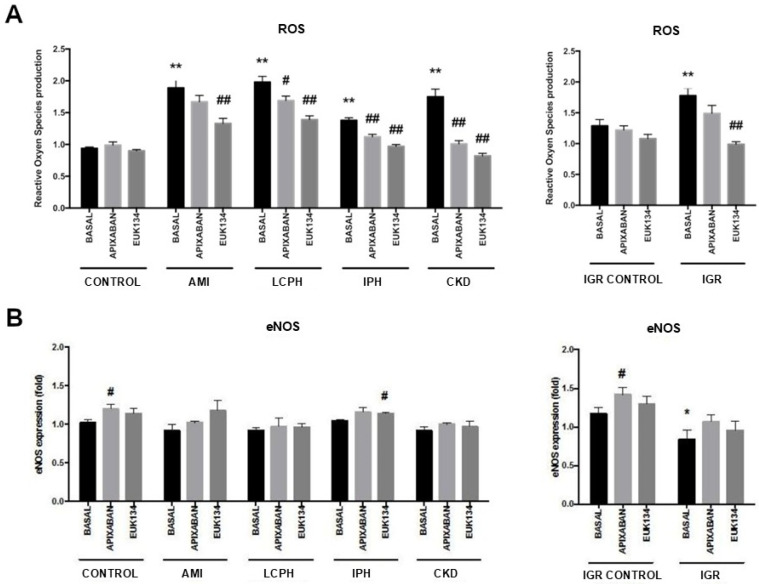
Bar diagrams representing (**A**) reactive oxygen species (ROS) production and (**B**) eNOS expression after exposing endothelial cells (ECs) to different serum samples: from healthy donors (CONTROL), and from patients who have coronary artery disease with acute myocardial infarction (AMI), liver cirrhosis with portal hypertension (LCPH), idiopathic pulmonary arterial hypertension (IPH), and chronic kidney disease (CKD) and pregnant women with intrauterine growth restriction (IGR). Results from the latest group were compared with those obtained with sera from a control group of pregnant women (IGR CONTROL). Studies were performed in the absence (BASAL) and presence of apixaban and EUK134. Results are expressed as fold increases with respect to those obtained by exposing cells to a commercial human serum (Mean ± SEM, *n* = 6). * *p* < 0.05 and ** *p* < 0.01 compared to the control group (CONTROL). # *p* < 0.05 and ## *p* < 0.01 compared to the respective basal state of each group (BASAL).

Changes in reactive oxygen species (ROS) production were detected and significantly elevated in all conditions as compared with the corresponding controls (Supplementary Table S1). Incubation with apixaban or EUK134 resulted in a decrease in ROS production in all study groups compared to the absence (Supplementary Table S2, Figure 5A).

### 2.4. eNOS Expression on ECs Exposed to Patients’ Sera

Levels of endothelial nitric oxide synthase (eNOS) in all conditions were similar to those of the CONTROL group, except for those in IGR which were significantly reduced compared to those in IGR CONTROL (Supplementary Table S1). In the presence of apixaban and EUK134, eNOS expression was increased in all study conditions compared to the absence (Supplementary Table S2, Figure 5B).

### 2.5. Identification of Serum Biomarkers of Endothelial Dysfunction

We initially considered 673 serum molecules for association with ED, including 400 metabolites and 273 proteins. After excluding 94 molecules that were absent in more than 80% of serum samples, 386 metabolites and 193 proteins were further investigated as potential biomarkers for ED. Supplementary Table S3 lists the 18 metabolites and 24 proteins with both an area under the receiver-operating characteristic curve (AUC-ROC) and hit rates over 77.5%, as well as the most discriminant cut-off values and the rate of positive determinations (above the cut-off) in the 66 cases with available data. Rates of positive values ranged from 10.5% for metabolite sphingomyelin (SM) 40:1 to 46.5% for apolipoprotein L1, with a median of 30.5%.

### 2.6. Serum Biomarkers and Clinical Prognosis

**Figure 6:**
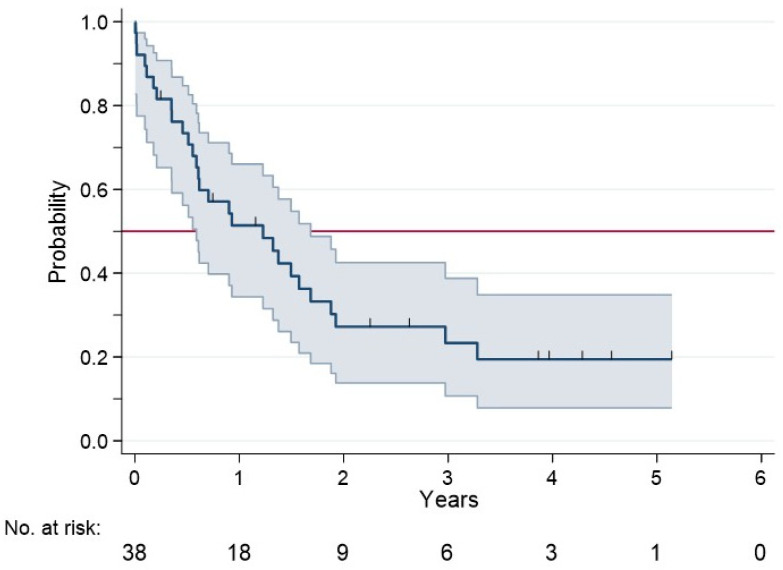
Event-free survival (EFS, 95% confidence interval) in 38 patients with chronic kidney disease (CKD) or liver cirrhosis with portal hypertension (LCPH). Blue line represents the Kaplan–Meier estimate of EFS for the study population. Red line marks the 50% EFS probability, allowing visual identification of the median EFS. Gray area indicates the 95% confidence interval of the EFS estimate, reflecting the statistical uncertainty around the survival probability at each time point.

**Figure 7:**
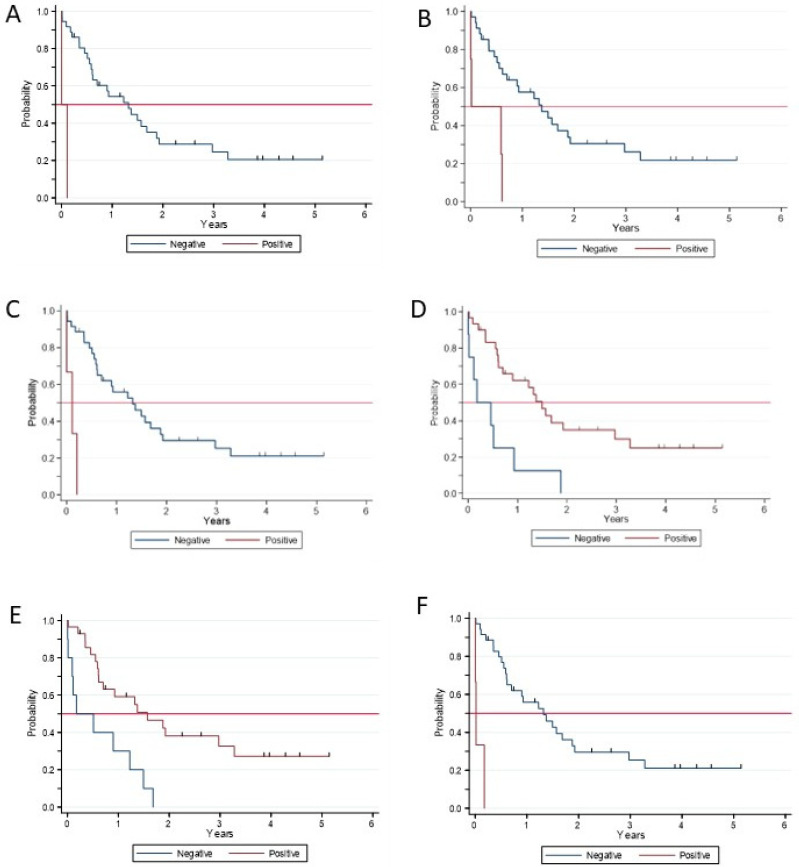
Event-free survival (EFS) in 38 patients with chronic kidney disease (CKD) or liver cirrhosis with portal hypertension (LCPH) according to serum biomarker levels of endothelial dysfunction (ED) above (“positive”) or below (“negative”) the optimal cut-off in brackets. Horizontal pink line marks the 50% EFS probability, allowing visual identification of the median EFS. (**A**) Sphingomyelin 40:1 (1.053), (**B**) sphingomyelin 40:2 (0.896), (**C**) sphingomyelin 42:1 (0.836), (**D**) 2-hydroxyglutaric acid (−0.323), (**E**) α-ketoglutaric acid (−0.275), (**F**) Insulin-like growth factor-binding protein complex acid-labile subunit (0.725).

These 42 selected biomarkers were further investigated for prognostic significance in a cohort of patients with CKD or LCPH (19 patients per group). We excluded patients with AMI or IPH because of the low rate of adverse events during the limited follow-up period. Supplementary Table S4 summarizes the main patient characteristics, and Figure 6 illustrates the cohort’s event-free survival (EFS). Six serum molecules were significantly associated with EFS. Positive values of SM 40:1, SM 40:2, SM 42:1, and Insulin-like growth factor-binding protein complex acid-labile subunit predicted a significantly shorter EFS, as also did negative values of 2-hydroxyglutaric acid and α-ketoglutaric acid (Table 1 and Figure 7).

## 3. Discussion

Endothelial dysfunction (ED) is a central pathological feature shared by a wide range of diseases associated with vascular dysfunction, despite their markedly different clinical presentations [22,23,24]. In the present study, we demonstrate that sera from patients with liver cirrhosis and portal hypertension (LCPH), chronic kidney disease (CKD), idiopathic pulmonary arterial hypertension (IPH), acute myocardial infarction (AMI), and intrauterine growth restriction (IGR) induce a common pattern of endothelial activation and damage in vitro. This phenotype is characterized by increased expression of adhesion molecules, enhanced oxidative stress, an elevated Von Willebrand Factor, and increased thrombogenicity of the extracellular matrix. Importantly, these alterations were consistently attenuated by anti-inflammatory and antioxidant interventions, supporting the concept that ED is a dynamic, potentially reversible process across diverse pathological conditions.

By exposing ECs to patient sera rather than disease-specific experimental stimuli, our approach captures the integrated effect of circulating inflammatory, metabolic, and oxidative factors present in each condition. Despite the heterogeneity of the diseases studied, ECs exhibited remarkably consistent responses, including increased expression of VCAM-1 and ICAM-1, enhanced ROS production, and a prothrombotic extracellular matrix. These findings suggest that systemic circulating factors converge on common endothelial signaling pathways, leading to a stereotyped activation profile irrespective of the underlying disease, as previously proposed in experimental and clinical studies of ED [25,26,27].

The myocardial infarction group showed a less pronounced increase in some adhesion markers and platelet adhesion. These findings may reflect differences between acute and chronic endothelial injury, the influence of background pharmacological treatments commonly used in coronary artery disease, or temporal variability in circulating mediators following myocardial infarction [10,28,29]. This observation highlights that, while ED is a shared mechanism, its magnitude and manifestations may differ across disease stages and clinical contexts.

Oxidative stress emerged as a consistent hallmark of endothelial activation across all conditions studied [30,31,32]. Therefore, oxidative stress and endothelial activation appear as central drivers. Increased ROS production was observed in ECs exposed to patients’ sera and was markedly reduced by the antioxidant EUK134 and by low-dose apixaban. The reduction in oxidative stress paralleled a decreased expression of adhesion molecules and reduced extracellular matrix thrombogenicity, underscoring the tight interplay between oxidative stress, inflammation, and endothelial prothrombotic remodeling [33,34].

Basal eNOS expression was not significantly altered in most disease groups, suggesting that early or intermediate stages of ED may be dominated by inflammatory and oxidative pathways rather than by overt impairment of nitric oxide synthase expression. This finding is consistent with previous observations indicating that functional uncoupling or reduced nitric oxide bioavailability may precede detectable changes in eNOS expression [35,36,37,38]. The increase in eNOS expression observed after pharmacological intervention may therefore reflect a restoration of endothelial homeostasis rather than a correction of a primary defect [39].

Beyond functional endothelial readouts, our integrated proteomic and metabolomic analyses identified a restricted panel of circulating molecules that discriminated with good accuracy between patients with ED and healthy controls. Among these, sphingomyelin species and proteins related to inflammatory and metabolic pathways were consistently altered across diseases. Importantly, a subset of these biomarkers was significantly associated with EFS in patients with LCPH and CKD, linking systemic endothelial alterations to clinically meaningful outcomes.

The association of sphingomyelins (SM 40:1, SM 40:2, SM 42:1) with shorter EFS suggests a role for lipid remodeling and membrane-related signaling in endothelial stress and disease progression, as previously suggested in conditions characterized by chronic inflammation and vascular dysfunction, with exceptions to the IGR groups [40,41,42,43]. Conversely, lower levels of metabolites related to mitochondrial metabolism, such as α-ketoglutaric acid and 2-hydroxyglutaric acid, were also associated with adverse outcomes, pointing to a potential link between endothelial metabolic resilience and clinical prognosis. While these associations do not establish causality, they are consistent with experimental data linking metabolic imbalance, oxidative stress, and endothelial injury [44,45].

Regarding the therapeutic implications and translational relevance of our findings, the attenuation of endothelial activation by both an anti-factor Xa agent and an antioxidant highlights the potential of targeting inflammatory and oxidative pathways to preserve endothelial function across different disease settings. Although apixaban was used here at low concentrations primarily as a proof-of-concept anti-inflammatory intervention rather than as an anticoagulant, its effects reinforce the close interconnection between coagulation, inflammation, and endothelial biology [19,46]. These findings are consistent with clinical evidence showing that reduction in vascular resistance, inflammation, or oxidative stress can improve outcomes in cirrhosis, chronic kidney disease, pulmonary hypertension, coronary artery disease, and preeclampsia [47,48,49,50,51,52,53].

Several limitations should be acknowledged. Using a single microvascular endothelial cell line does not capture the full heterogeneity of endothelial phenotypes across vascular beds, which differ by anatomical location and local environmental cues [54,55]. The prognostic analyses were performed in relatively small cohorts and should therefore be interpreted as hypothesis-generating. Validation of the identified biomarkers in larger, independent cohorts and mechanistic studies addressing their functional role in endothelial biology is required.

In conclusion, this study demonstrates that ED constitutes a unifying biological mechanism across multiple high-risk diseases and reflects a shared systemic response to inflammatory, oxidative, and metabolic stressors. By combining functional endothelial assays with integrated proteomic and metabolomic profiling, we identified common circulating biomarkers associated with endothelial activation and event-free survival, highlighting their potential value for vascular risk stratification across different disease settings. Together, these findings support the concept of endothelial dysfunction as a central, dynamic, and potentially reversible driver of disease progression, and provide a strong rationale for the development of integrated diagnostic and therapeutic strategies aimed at restoring endothelial homeostasis.

## 4. Materials and Methods

### 4.1. Experimental Design

Endothelial cells (ECs) in culture were grown in media supplemented with 20% human serum from the patients under study and controls (*n* = 20 patients/group). A control group (CONTROL) consisted of samples from healthy donors matched by sex and age. Also, a commercial control serum (Sigma-Aldrich Quimica SA, Madrid, Spain) was used in the in vitro experiments. Results obtained with patients’ and control individuals’ sera were always expressed as fold increases with respect to the commercial sera. Results from the IGR group were compared with those from pregnant women without IGR (IGR CONTROL).

Experiments were performed in the absence and presence of an anti-factor Xa agent, apixaban (Bristol Myers-Squibb, New York, NY, USA), and a synthetic catalase/superoxide dismutase (SOD) mimetic, EUK134 (Cayman Chemical, Ann Arbor, MI, USA). The apixaban dose (60 ng/mL) was selected from a study by Frost [56] as representative of patients on low-dose apixaban (close to the mean Cmax) and was used in a previous study [19]. The dose of EUK134 (1 uM) was selected from a previous study by our group [21]. A cell viability assay (MTT) was performed to ensure the lack of toxicity.

Experiments were performed to evaluate changes in the expression of VCAM-1, ICAM-1, eNOS, VWF, and reactive oxygen species (ROS) production by immunofluorescence, and the reactivity of the extracellular matrix (ECM) towards circulating platelets was analyzed using blood perfusion techniques (*n* = 6 for in vitro experiments). Proteomic and metabolomic assays were performed to identify serum biomarkers of ED. Expected outcomes of the present study were: (a) identification of circulating molecules, including metabolites and proteins, which accurately discriminate ED; and (b) evaluation of the association of previous biomarkers with prognostic EFS rates.

### 4.2. Patients and Sample/Data Collection

Sera samples were collected during the recruiting period (from 2016 to 2018) from patients and controls, centrifuged (3000× *g*, 15 min), aliquoted, and immediately stored at −40 °C until analysis. Patients with other conditions besides the ones described above were excluded. All patients provided written informed consent to participate in the study, and the study was approved by the Hospital Clinic Ethical Committee for Clinical Investigation (HCB_2015-0585).

All patients included in the present study were diagnosed with their respective pathologies in accordance with the established clinical guidelines for each condition and followed by their clinicians at our institution.

### 4.3. Endothelial Cell Cultures

A human microvascular endothelial cell line (HMEC-1, ATCC, Manassas, VA, USA) was used. ECs were maintained at 37 °C in a 5% CO_2_ humidified incubator and grown in media MCDB 131 (Gibco BRL, ThermoFisher, Waltham, MA, USA) supplemented with 10 mM of glutamine (Gibco BRL, ThermoFisher), 100 U/mL of penicillin, 100 μg/mL of streptomycin (Gibco BRL, ThermoFisher) 1 μg/mL of hydrocortisone (Sigma-Aldrich Quimica SA) and 10 ng/mL of epidermal growth factor (EGF) (BD Bioscience, Erenbodegem, Belgium) with 20% human sera under study. Culture media were replaced 3 times a week. Cells were grown on 8-well μ-Slides (#80826, Ibidi GmbH, Planegg, Germany) or on 1% gelatin-coated 18 mm × 18 mm glass coverslips.

### 4.4. Immunofluorescence Evaluation of Endothelial Biomarkers: VCAM-1, ICAM-1, VWF and eNOS

Ibidi 8-well μ-Slides were seeded with ECs exposed to the sera or treatment in study for 72 h. Then, confluent cell monolayers ere fixed in 4% paraformaldehyde (10 min RT), blocked with 1% BSA in PBS for 45 min and incubated with the appropriate antibodies: ICAM-1 (sc-107, Santa Cruz Biotechnology, Dallas, TX, USA; dilution 1:100, 1 h RT), VCAM-1 (GTX 110684, GeneTex International Corporation, Hsinchu City, Taiwan; dilution 1:100, 1 h RT), eNOS (sc-376751, Santa Cruz Biotechnology; dilution 1:100, 1 h RT) and VWF (A0082, Dako, Agilent Technologies, Santa Clara, CA, USA; dilution 1:2000, 1 h RT).

Anti-rabbit or anti-mouse secondary antibodies (dilution 1:500 or 1:2000, respectively, for 1 h at RT) conjugated with Alexa 488, 555, or 594 (Molecular Probes, New York, NY, USA) were used. Samples were evaluated by light microscopy (Leica DM4000B, Barcelona, Spain): 15 microscopic fields from each condition were captured through a video camera (Leica DFC301FX, Barcelona, Spain), and the density of labeling was assessed by computerized morphometric analysis (ImageJ Fiji 1.x, National Institutes of Health, Bethesda, MD, USA). Results were expressed as fold increases (Mean ± SEM) in the surface covered by fluorescent stain, for VCAM-1 and ICAM-1, and the mean fluorescence intensity for VWF and eNOS, with respect to control experiments.

### 4.5. Reactivity of the Extracellular Matrix Towards Platelets

ECs seeded in 1% gelatin-coated coverslips were grown to confluence for 6–8 days. Then, cells were extracted with 3% EGTA for 1 h at 37 °C, and washed with PBS to obtain the ECM. Perfusion studies using citrated blood from healthy donors, using a parallel-plate perfusion chamber (shear rate of 800 s^−1^ for 5 min). Then, coverslips were washed with PBS, fixed in 0.5% glutaraldehyde for 24 h at 4 °C, and stained with 0.02% toluidine blue. The reactivity of platelet towards ECM was evaluated in 15 microscopic fields per condition, and results were expressed as fold increases in platelet-covered surface (Mean ± SEM) with respect to controls, as previously described.

### 4.6. Quantification of Intracellular Reactive Oxygen Species (ROS)

To quantify the oxidative state of cell cultures and treatments, ECs were seeded in 96-well plates (Labclinics, Barcelona, Spain). Then, cells were incubated with 5- (and 6)-chloromethyl-2′, 7′-dichlorodihydrofluorescein diacetate, and acetyl ester (CM-H2DCFDA) (#C6827; Molecular Probes, Eugene, OR, USA) at a final concentration of 15 uM for 30 min, washed with PBS, and exposed to media containing the sera or treatments under study for 4 h at 37 °C. Fluorescence was captured through a microplate reader (Tekan Infinite 200 PRO, Männedorf, Switzerland).

### 4.7. Proteomic Analysis

Before proteomic analysis, the depletion of the seven most abundant serum proteins (Albumin, Immunoglobulin G, antitrypsin, Immunoglobulin A, transferrin, haptoglobin, and fibrinogen) was performed to increase the number of identified proteins [57]. The methodology applied for proteomic analysis is described in previous studies performed by the group [58].

### 4.8. Metabolomic Analysis

The metabolomic analysis included the analysis of lipids (lipidomics), amino acids, and polar metabolites, performed through a well-established four-level extraction protocol. Lipidomic analysis was performed using two methods: methanol extraction and chloroform:methanol extraction (Folch method). Methodology used is detailed in previous studies by the group [59]. The analysis was performed by investigators blinded to the study group of each sample.

### 4.9. Statistical Methods

Kolmogorov–Smirnov or Shapiro–Wilk normality tests were applied for each continuous variable, depending on the *n*. For the in vitro experiments with ECs, data are expressed as fold increases in Mean ± SEM for normally distributed continuous variables (*n* = 6). Data from experiments performed with samples from all the studied groups followed a normal distribution, and were analyzed by Student’s unpaired *t*-test, comparing each group vs. the control group, or vs. the basal state (for those studies with drugs). All statistical analyses were conducted using GraphPad Prism, version 4.02. *p* < 0.05 was considered significant.

To identify serum biomarkers of ED potentially useful in clinical practice, the molecular yield of proteomic and metabolomic studies was compared between patients whose serum induced ED in vitro and their corresponding controls.

Area under the receiver-operating characteristic curve (AUC-ROC) and hit rates were derived from logistic regression of biomarker levels to cases (*n* = 77) and controls (*n* = 28). Since there was substantial variation among biomarkers in measurement units and ranges, serum concentrations or activities were standardized to units of standard deviation around the mean. Preliminary selection of potential biomarkers for ED was based on attaining both an AUC-ROC and a hit rate greater than 77.5%. Hit rates were calculated as the quotient of true positives plus true negatives to the total number of cases. The 77.5% threshold was arbitrarily selected as the most appropriate to avoid premature elimination of potentially useful biomarkers while minimizing the false-positive rate. Standardized values were then categorized as “positive” (over the cut-off) or “negative” (below the cut-off) for their association with ED after having determined the optimal cut-off by using Liu’s method [60]. Based on this method, the optimal cut-off value corresponds to the point that maximizes the product of sensitivity and specificity.

The main clinical outcome was event-free survival (EFS), measured in years from the date of study entry to diagnosis of ascites, bleeding from esophageal varices, portal thrombosis, liver or kidney transplant, or death from any cause, whichever occurred first. Patients were followed for a period of 5 years for the EFS analysis. The association of the selected biomarkers with EFS was evaluated using Cox regression and graphically represented using the Kaplan–Meier method. Clinical outcomes were measured only in patients with CKD or LCPH because of the low rate of adverse events in patients with AMI or IPH, given the relatively short follow-up.

## Figures and Tables

**Table 1:** Serum biomarkers of endothelial dysfunction (ED) associated with shortened event-free survival (EFS) in 38 patients with chronic kidney disease (CKD) or liver cirrhosis and portal hypertension (LCPH).

Biomarker	Hazard Ratio	*p*
Sphingomyelin (40:1)	20.1 (3.3–122)	0.001
Sphingomyelin (40:2)	5.3 (1.7–17.1)	0.005
Sphingomyelin (42:1)	14.4 (3.1–64.5)	0.001
2-hydroxyglutaric acid	0.26 (0.11–0.62)	0.002
α-ketoglutaric acid	0.28 (0.12–0.64)	0.003
Insulin-like growth factor-binding protein complex acid-labile subunit	22.4 (4.4–114)	<0.001

## Data Availability

Data is unavailable due to privacy and ethical restrictions.

## Supplementary Materials

The following supporting information can be downloaded at https://www.mdpi.com/article/10.3390/ijms27093829/s1.

## Abbreviations

The following abbreviations are used in this manuscript:

AMI	Acute myocardial infarction
APIX	Apixaban
AUC-ROC	Area under the receiver-operating characteristic curve
CKD	Chronic kidney disease
ECM	Extracellular matrix
ECs	Endothelial cells
ED	Endothelial dysfunction
EFS	Event-free survival
eNOS	Endothelial nitric oxide synthase
EUK134	Synthetic superoxide dismutase (SOD)/catalase mimetic
ICAM-1	Intercellular adhesion molecule 1
IGR	Intrauterine growth restriction
IPH	Idiopathic pulmonary arterial hypertension
LCPH	Liver cirrhosis and portal hypertension
ROS	Reactive oxygen species
SM	Sphingomyelin
SOD	Superoxide dismutase
VCAM-1	Vascular cell adhesion protein 1
VWF	Von Willebrand Factor
