# Supplementary material for: Common Biomarkers of Endothelial Dysfunction Across Highly Prevalent Diseases with Cardiovascular Risk: Functional Characterization and Prognostic Implications
Source: Int J Mol Sci. 2026 Apr 25;27(9):3829. doi: 10.3390/ijms27093829 (PMC13164163; doi:10.3390/ijms27093829)
Supplement: Supplementary file 1 [file ijms-27-03829-s001.zip › ijms-4224222-supplementary.pdf]

Supplementary Materials

Table S1. Biomarkers at basal state expressed in fold increase with respect to commercial control serum (Mean ± SEM, n = 6 for each biomarker).

	CONTROL	AMI	LCPH	IPH	CKD	IGR CONTROL	IGR
**VCAM-1**	1.7 ± 0.3	3.0 ± 0.8	5.9 ± 1.1 **	7.6 ± 0.5 **	7.7 ± 1.0 **	2.4 ± 0.8	8.9 ± 0.3 *
**ICAM-1**	1.3 ± 0.1	3.0 ± 0.4 **	1.7 ± 0.2 *	1.9 ± 0.1 **	1.6 ± 0.1 *	1.4 ± 0.2	2.1 ± 0.5
**VWF**	1.1 ± 0.1	2.2 ± 0.3 *	3.4 ± 0.3 **	1.5 ± 0.1 *	2.0 ± 0.1 **	1.6 ± 0.2	3.4 ± 0.6 **
**PLATELET ADHESION**	1.0 ± 0.1	1.5 ± 0.3	1.3 ± 0.0 *	1.4 ± 0.0 *	1.5 ± 0.1 **	0.8 ± 0.0	1.4 ± 0.1 **
**ROS**	0.9 ± 0.0	1.9 ± 0.2 **	2.0 ± 0.1 **	1.4 ± 0.1 **	1.8 ± 0.1 **	1.3 ± 0.1	1.8 ± 0.1 **
**eNOS**	1.0 ± 0.0	0.9 ± 0.0	0.9 ± 0.0	1.0 ± 0.0	0.9 ± 0.1	1.2 ± 0.1	0.8 ± 0.1 *

* $p < 0.05$ and ** $p < 0.01$ compared to the control group, consisting of healthy donors. AMI: coronary artery disease with acute myocardial infarction; LCPH: liver cirrhosis with portal hypertension; IPH: idiopathic pulmonary arterial hypertension; CKD: chronic kidney disease; IGR: placental disorders such as intrauterine growth restriction (IGR). APIX: apixaban; EUK134: synthetic catalase/superoxide dismutase (SOD) mimetic.

Table S2. Biomarkers in the presence of apixaban (APIX) and EUK134 expressed in fold increase with respect to commercial control serum (Mean ± SEM, n = 6 for each biomarker).

	CONTROL		AMI		LCPH		IPH		CKD		IGR CONTROL		IGR	
	APIX	EUK 134	APIX	EUK 134	APIX	EUK 134	APIX	EUK 134	APIX	EUK 134	APIX	EUK 134	APIX	EUK 134
**VCAM-1**	1.0 ± 0.2	1.4 ± 0.3	0.8 ± 0.2 *	0.7 ± 0.1 *	2.4 ± 0.2 **	2.4 ± 0.5 **	2.8 ± 0.5 **	2.9 ± 0.2 **	1.4 ± 0.3 **	1.6 ± 0.4 **	1.2 ± 0.3	0.9 ± 0.4	2.5 ± 0.2 **	3.9 ± 0.2 **
**ICAM-1**	1.0 ± 0.1	0.8 ± 0.1 *	1.2 ± 0.0 *	1.3 ± 0.3 *	1.1 ± 0.0 *	1.2 ± 0.0 *	1.4 ± 0.0 **	1.2 ± 0.1 **	1.1 ± 0.2 *	0.7 ± 0.0 **	1.3 ± 0.3	0.8 ± 0.1 *	1.3 ± 0.1 *	1.3 ± 0.3 *
**VWF**	1.0 ± 0.2	0.8 ± 0.1 *	1.4 ± 0.1 *	1.2 ± 0.2 *	2.8 ± 0.2 *	2.8 ± 0.2 *	1.1 ± 0.1 *	1.1 ± 0.0 *	1.3 ± 0.0 **	1.4 ± 0.1 *	1.2 ± 0.1 *	1.3 ± 0.1	2.5 ± 0.2	2.1 ± 0.4 *
**PLATELET ADHESION**	0.8 ± 0.2	0.7 ± 0.2 *	1.1 ± 0.2 *	1.3 ± 0.2	1.1 ± 0.1	0.8 ± 0.1 *	1.2 ± 0.1 *	1.2 ± 0.0 **	1.1 ± 0.1 *	1.1 ± 0.1 *	0.7 ± 0.1	0.4 ± 0.0 *	1.2 ± 0.1 *	1.2 ± 0.1 *
**ROS**	1.0 ± 0.1	0.9 ± 0.0	1.7 ± 0.1	1.3 ± 0.1 **	1.7 ± 0.1 *	1.4 ± 0.1 **	1.1 ± 0.1 **	1.0 ± 0.1 **	1.0 ± 0.1 **	0.8 ± 0.0 **	1.2 ± 0.1	1.1 ± 0.1	1.5 ± 0.1	1.0 ± 0.0 **
**eNOS**	1.2 ± 0.1 *	1.1 ± 0.1	1.1 ± 0.1	1.2 ± 0.1	1.0 ± 0.1	1.0 ± 0.1	1.2 ± 0.1	1.1 ± 0.0 *	1.0 ± 0.0	1.0 ± 0.1	1.4 ± 0.1 *	1.3 ± 0.1	1.1 ± 0.1	1.0 ± 0.1

* $p < 0.05$ and ** $p < 0.01$ compared to the respective basal state of each group, shown in Supplemental Table 1. AMI: coronary artery disease with acute myocardial infarction; LCPH: liver cirrhosis with portal hypertension; IPH: idiopathic pulmonary arterial hypertension; CKD: chronic kidney disease; IGR: placental disorders such as intrauterine growth restriction (IGR); APIX: apixaban; EUK134: synthetic catalase/superoxide dismutase (SOD) mimetic.

Table S3. Serum metabolites and proteins (shadowed table cells) selected as potential biomarkers of endothelial dysfunction (ED).

Biomarker	AUC-ROC (%)	Hits rate (%)	Best cut-off	No. > cut-off	No. ≤ cut-off	Positive rate (%)
Anserine	84.87	77.27	-0.121	32	34	33.5
Cystathione	94.55	83.33	-0.337	40	26	41.5
Gamma Aminobutyric acid	88.44	77.27	-0.173	28	38	29.5
Hydroxylisine	89.47	81.82	-0.504	42	24	43.5
Hydroxyproline trans	88.53	80.30	-0.242	31	35	32.5
Kynurenine	86.47	80.30	-0.449	37	29	38.5
Sphingomyelin (39:1)	86.84	81.82	0.628	18	48	19.5
Sphingomyelin (40:1)	93.89	87.88	1.053	9	57	10.5
Sphingomyelin (40:2)	84.12	75.76	0.896	13	53	14.5
Sphingomyelin (41:1)	88.06	77.27	0.564	19	47	20.5
Sphingomyelin (42:1)	90.32	84.85	0.836	13	53	14.5
Acetyl-carnitine	85.90	83.33	-0.384	33	33	34.5
Dehydroepiandrosterone sulfate	83.18	78.79	-0.516	41	25	42.5
Taurocholic acid	81.02	75.76	-0.401	26	40	27.5
Taurodeoxycholic acid	78.67	77.27	-0.319	26	40	27.5
2-hydroxyglutaric acid	82.80	83.33	-0.323	33	33	34.5
Alpha-Ketoglutaric acid	84.12	78.79	-0.275	29	37	30.5
Sucrose	89.10	81.82	-0.479	34	32	35.5
Inter-alpha-trypsin inhibitor heavy chain 1	83.36	75.76	0.199	33	33	34.5
Inter-alpha-trypsin inhibitor heavy chain 2	87.69	84.85	0.024	36	30	37.5
Plasminogen	86.75	77.27	0.082	29	37	30.5
Apolipoprotein A-II	88.72	75.76	0.312	26	40	27.5
Apolipoprotein A-IV	86.28	83.33	-0.150	29	37	30.5
Thyroid hormone receptor beta	87.50	80.30	0.883	12	54	13.5
Complement component C7	82.42	75.76	-0.410	26	40	27.5
Alpha-2-antiplasmin	93.14	83.33	0.378	22	44	23.5
Insulin-like growth factor-binding protein complex acid-labile subunit	93.42	83.33	0.725	20	46	21.5
Clusterin	78.29	78.79	0.771	15	51	16.5
Lumican	88.63	75.76	-0.253	27	39	28.5

Pigment epithelium-derived factor	93.42	84.85	-0.129	33	33	34.5
Coagulation factor XII	77.35	75.76	-0.569	44	22	45.5
Carboxypeptidase N subunit 2	82.71	75.76	0.555	20	46	21.5
Von Willebrand factor	85.24	80.30	-0.374	33	33	34.5
Complement component C8 beta chain	82.80	77.27	0.580	18	48	19.5
Apolipoprotein M	86.98	78.79	0.017	30	36	31.5
Apolipoprotein L1	84.49	80.30	-0.445	45	21	46.5
Lipopolysaccharide-binding protein	77.54	75.76	-0.351	38	28	39.5
Fibulin-1	83.18	78.79	-0.101	26	40	27.5
Lysozyme C	85.71	75.76	-0.484	37	29	38.5
CD44 antigen	96.05	90.91	-0.368	35	31	36.5
Complement factor D	82.71	78.79	-0.208	34	32	35.5
Cytochrome c	83.41	77.27	-0.152	29	37	30.5

Table S4. Clinical characteristics of 38 patients with chronic kidney disease (CKD) or liver cirrhosis and portal hypertension (LCPH) were evaluated for biomarkers of endothelial dysfunction (ED).

**Age***	61 (50-71)
**Sex. men/women**	24 (63%)/14
**Smokers**	17 (45%)
**Diabetes**	3 (8%)
**Arterial hypertension**	18 (47%)
**Obesity (BMI > 30)**	12 (32%)
**High serum LDL**	11 29%)
**eGFR (ml/min/m^2)***	31.5 (15.0-90.0)
**≥ 60**	18 (47%)
**< 30**	19 (50%)

* Median and interquartile range. LDL: low-density lipoproteins; eGFR: estimated glomerular filtration rate.
